# Lower Dexamethasone Dose in Multiple Myeloma: A Multicenter Retrospective Analysis

**DOI:** 10.3390/cancers18213565

**Published:** 2026-11-06

**Authors:** Lili Korpás, Ádám Wiedemann, Fruzsina Szél, Szabolcs Svorenj, Júlia Weisinger, Zsolt Nagy, Zsuzsanna Szemlaky, Zoltán Csukly, Péter Reményi, Péter Rajnics, Elvira Altai, Márk Plander, Krisztina Kórád, Erika Szaleczky, András Masszi, Szabolcs Kosztolányi, Hussain Alizadeh, Szabolcs Modok, Zita Borbényi, Szilvia Lovas, László Váróczy, Tamás Masszi, Gábor Mikala, Gergely Varga

**Affiliations:** 1 Department of Internal Medicine and Haematology, Semmelweis University, 1085 Budapest, Hungary; korpaslili.coordimed@gmail.com (L.K.); wiedemannadam@yahoo.com (Á.W.); fruzsinaszel@gmail.com (F.S.); svorenj.szabolcs@gmail.com (S.S.); weisingerjulia@gmail.com (J.W.); nagy.zsolt@semmelweis.hu (Z.N.); masszi.tamas@semmelweis.hu (T.M.); mikala.gabor@semmelweis.hu (G.M.); 2 Department of Hematology and Stem Cell Transplantation, South Pest Central Hospital, National Institute for Hematology and Infectious Diseases, 1097 Budapest, Hungary; szemlaky.zsuzsanna@dpckorhaz.hu (Z.S.); csukly.zoltan@dpckorhaz.hu (Z.C.); premenyi@dpckorhaz.hu (P.R.); 3 Somogy County Kaposi Mór Teaching Hospital, 7400 Kaposvár, Hungary; rajnicsp@hotmail.com; 4 Hematology, Veszprém-Csolnoky Ferenc County Hospital, 8200 Veszprém, Hungary; altaielvira@gmail.com; 5 Markusovszky University Teaching Hospital, 9700 Szombathely, Hungary; planderm@yahoo.com; 6 Borsod-Abaúj-Zemplén Central County University Teaching Hospital, 3526 Miskolc, Hungary; koradkrisztina@hotmail.com; 7 National Institute of Oncology, 1122 Budapest, Hungary; szaleczky.erika@oncol.hu (E.S.); masszi.andras@oncol.hu (A.M.); 8 1st Department of Internal Medicine, University of Pécs, 7624 Pécs, Hungary; kosztolanyi.szabolcs@pte.hu (S.K.); alizadeh.hussain@gmail.com (H.A.); 9 2nd Department of Medicine and Cardiology Center, University of Szeged, 6720 Szeged, Hungary; moszadokbolcs@gmail.com (S.M.); borbenyizita@gmail.com (Z.B.); 10 Department of Hematology, Institute for Medicine, Clinical Center, University of Debrecen, 4032 Debrecen, Hungary; lovasszilvia09@gmail.com (S.L.); laszlo.varoczy@gmail.com (L.V.)

**Keywords:** multiple myeloma, lenalidomide-dexamethasone, dexamethasone dose reduction

## Abstract

**Background/Objectives:** Dexamethasone has long served as the cornerstone of therapy for multiple myeloma; however, its optimal dosing remains ill-defined, particularly when balanced against its profound toxicity profile. Recent therapeutic advances have consequently intensified interest in steroid dose-reduction strategies. This retrospective, multicenter study aimed to evaluate the impact of varied dexamethasone dosing regimens on clinical outcomes within a real-world setting. **Methods:** We conducted a retrospective multicenter study across 11 Hungarian centers, enrolling 272 patients with multiple myeloma who received lenalidomide–dexamethasone therapy. Treating physicians determined dosing strategies at their discretion for each patient. We retrospectively stratified patients into three cohorts based on their prescribed weekly dexamethasone dose: 40 mg, 20 mg, and less than 20 mg. **Results:** We observed a distinct interaction between patient age and weekly dexamethasone dose. Nonetheless, we detected no statistically significant difference in progression-free survival (PFS) among the three dosing cohorts (*p* = 0.632). Similarly, dexamethasone dose intensity demonstrated no significant association with overall treatment response (*p* = 0.560). Subgroup analysis restricted to patients with high-risk cytogenetics also showed no significant disparities in PFS (*p* = 0.312). Furthermore, early treatment response rates at 2 and 6 months were also independent of the dexamethasone dose. Notably, grade 3–5 adverse events were numerically more frequent in the 40 mg group. The lack of significant differences in these outcome measures is particularly important, given the baseline age imbalances among the cohorts. **Conclusions:** Lower initial dexamethasone dose was not associated with statistically significant differences in progression-free survival or treatment response in this retrospective cohort. Although Grade 3–5 adverse events were numerically more frequent in the 40 mg group, these observations warrant further investigation in prospective studies.

## Simple Summary

Corticosteroids are an integral part of all currently used myeloma protocols; however, higher steroid doses can cause side effects. We examined whether lower starting doses of dexamethasone could be used without reducing treatment effectiveness. In this retrospective multicenter study, we analyzed 272 patients treated with lenalidomide and dexamethasone at 11 Hungarian centers. Patients received 40 mg, or reduced dose, 20 mg, or less than 20 mg dexamethasone weekly. Older patients more often received the lower dose. We did not find significant differences between the three groups in progression-free survival, overall treatment response, early treatment response, or adverse events. Our results suggest that lower dexamethasone doses may be used without reducing treatment efficacy. Further prospective studies are needed to confirm these findings.

## 1. Introduction

Multiple myeloma is a malignant plasma cell dyscrasia characterized by the clonal proliferation of plasma cells within the bone marrow microenvironment. Its pathognomonic clinical features include bone pain, pathological fractures, bone marrow failure, progressive renal impairment, and recurrent infections. Predominantly a disease of the elderly, the median age at diagnosis ranges between 66 and 70 years, with fewer than 2% of patients diagnosed under the age of 40. Multiple myeloma accounts for approximately 1% to 2% of all malignancies, with a global incidence rate of 4 to 5 cases per 100,000 person-years [1]. In Hungary, data from the National Cancer Registry documented 603 newly diagnosed cases in 2023 [2].

Dexamethasone has long served as a cornerstone of anti-myeloma therapy; however, its clinical prominence has shifted since the introduction of novel agents, including immunomodulatory drugs (IMiDs), proteasome inhibitors (PIs), monoclonal antibodies, and T-cell engagers.

While this potent corticosteroid was originally used as monotherapy or in combination with conventional chemotherapy, incorporating these newer targeted agents yielded substantial improvements in patient outcomes [3,4,5]. The adverse event profile of long-term or high-dose corticosteroid therapy is well-documented, encompassing an increased risk of severe infections, osteoporosis, new-onset or worsening diabetes mellitus, hypertension, and neuropsychiatric disturbances [6]. Furthermore, with the advent of immune-based therapeutic approaches, concerns have emerged about whether the lympholytic and immunosuppressive properties of dexamethasone might attenuate the efficacy of regimens that rely on the host immune system for their anti-myeloma effect. Consequently, the optimal dosing strategy for dexamethasone remains a subject of ongoing debate, particularly regarding the balance between maintaining therapeutic efficacy and minimizing systemic toxicity.

Historical therapeutic regimens heavily relied on high-dose dexamethasone—typically administered at 40 mg daily on a four-days-on, four-days-off schedule [7]. Pivotal clinical trials later showed that a de-escalated schedule of 40 mg once weekly significantly reduced systemic toxicity and improved overall survival [8]. Consequently, current clinical practice routinely incorporates further age-dependent dose modifications, typically reducing the weekly dose to 20 mg for patients aged 75 years or older [9,10,11,12]. The phase III randomized FIRST trial also evaluated the impact of age on outcomes with lenalidomide and low-dose dexamethasone in newly diagnosed multiple myeloma [13].

Accumulating evidence suggests that reducing dexamethasone doses maintains therapeutic efficacy while substantially improving tolerability, particularly in elderly patients or those with significant comorbidities [14]. Notably, a randomized study conducted by a Turin-based research group involving 210 patients evaluated the clinical impact of completely discontinuing dexamethasone after nine months of lenalidomide-based therapy. The experimental arm demonstrated non-inferior efficacy, that dexamethasone discontinuation after induction may be a feasible approach in selected patients [15]. Although numerous clinical centers have adapted their protocols to reflect these findings, this strategy has not yet been formally integrated into international guidelines. Similarly, the optimal initial dexamethasone dose remains an open question in clinical oncology.

Given these clinical challenges, many practitioners routinely prescribe corticosteroid doses below those recommended in standard guidelines [16]. However, the absence of robust, evidence-based protocols for systematic dose reduction remains a substantial challenge in routine clinical management.

To address this critical gap in the literature, the present study sought to generate real-world evidence evaluating the hypothesis that de-escalated initial dexamethasone dosing does not compromise therapeutic efficacy. Specifically, we aimed to assess the clinical impact of varied different dexamethasone dose levels on treatment outcomes within a real-world patient cohort.

## 2. Materials and Methods

### 2.1. Patients

We conducted a retrospective multicenter study including 283 patients with multiple myeloma from 11 centers who received lenalidomide–dexamethasone (RD) therapy as second- or later-line therapy, with one to four prior lines, between 2010 and 2020 for relapsed multiple myeloma [17]. Dexamethasone dose information was available in 272 cases, which were included in the final analysis. We collected fluorescence in situ hybridization (FISH) and International Staging System (ISS) data according to International Myeloma Working Group (IMWG) criteria. High-risk abnormalities were defined as t(4;14), t(14; 16) and/or del (17p) and 1q amplification [18].

We stratified patients into three groups based on the starting dexamethasone dose: <20 mg (*n* = 40), 20 mg (*n* = 100), and 40 mg (*n* = 132). Subsequent dexamethasone dose reductions or discontinuations during treatment were not recorded and could therefore not be evaluated in this study.

We analyzed progression-free survival (PFS), overall response rates, and cytogenetic risk. Median follow-up time was estimated using the reverse Kaplan–Meier method [19]. We obtained clinical and laboratory data from medical records. The primary endpoint of the study was PFS, while secondary endpoints included overall response and early response at 2 and 6 months.

### 2.2. Response Categories and Statistics

The overall response rate was defined as the collective proportion of patients with complete response (CR), very good partial response (VGPR) or partial response (PR) as their best response [20]. PFS was defined as the time from treatment start to disease progression or death. For AE grading we used CTCAE v5.0.

We performed statistical analyses using SPSS software (version 20.0; SPSS Inc., Chicago, IL, USA). Categorical variables were compared using the chi-square and Fisher’s exact tests, while continuous variables were analyzed using the Mann–Whitney U test and Spearman correlation analysis. We estimated PFS using the Kaplan–Meier method and compared differences between dose groups using the log-rank test. We used multivariate Cox proportional hazards regression to identify independent prognostic factors. A *p*-value < 0.05 was considered statistically significant.

## 3. Results

### 3.1. Patient Characteristics

A total of 283 patients were identified who were treated with RD, of whom 272 had available dexamethasone starting dose data and were therefore included in the analysis (Table 1). The majority of these, 133 patients, had one, 76 had two, and 63 had three or more prior lines. A total of 123 patients had prior autologous stem cell transplant (ASCT). FISH data were only available in 179 patients, of whom 42 had high-risk cytogenetics; ISS stage was available for 231 of 272 patients. The percentage of missing ISS and FISH results were similar in the three dex dose cohorts.

Baseline characteristics differed across the three starting dexamethasone dose groups, particularly with respect to age and prior ASCT. The proportion of patients with prior ASCT was significantly higher in the 40 mg group (58.8%) than in the 20 mg (35.0%) and <20 mg (27.5%) groups (*p* < 0.001). No statistically significant differences were observed in high-risk cytogenetics or renal impairment, although clinically relevant imbalances cannot be excluded.

### 3.2. Dexamethasone Dose, Age, and Treatment Response

As anticipated, older patients received lower baseline doses of dexamethasone, aligning with standard protocol recommendations that advise a dose reduction to 20 mg weekly for patients aged 75 years or older. Notably, however, a substantial proportion of patients initiated therapy at an even lower dose intensity (<20 mg/week, typically 8 mg or 12 mg).

**Figure 1:**
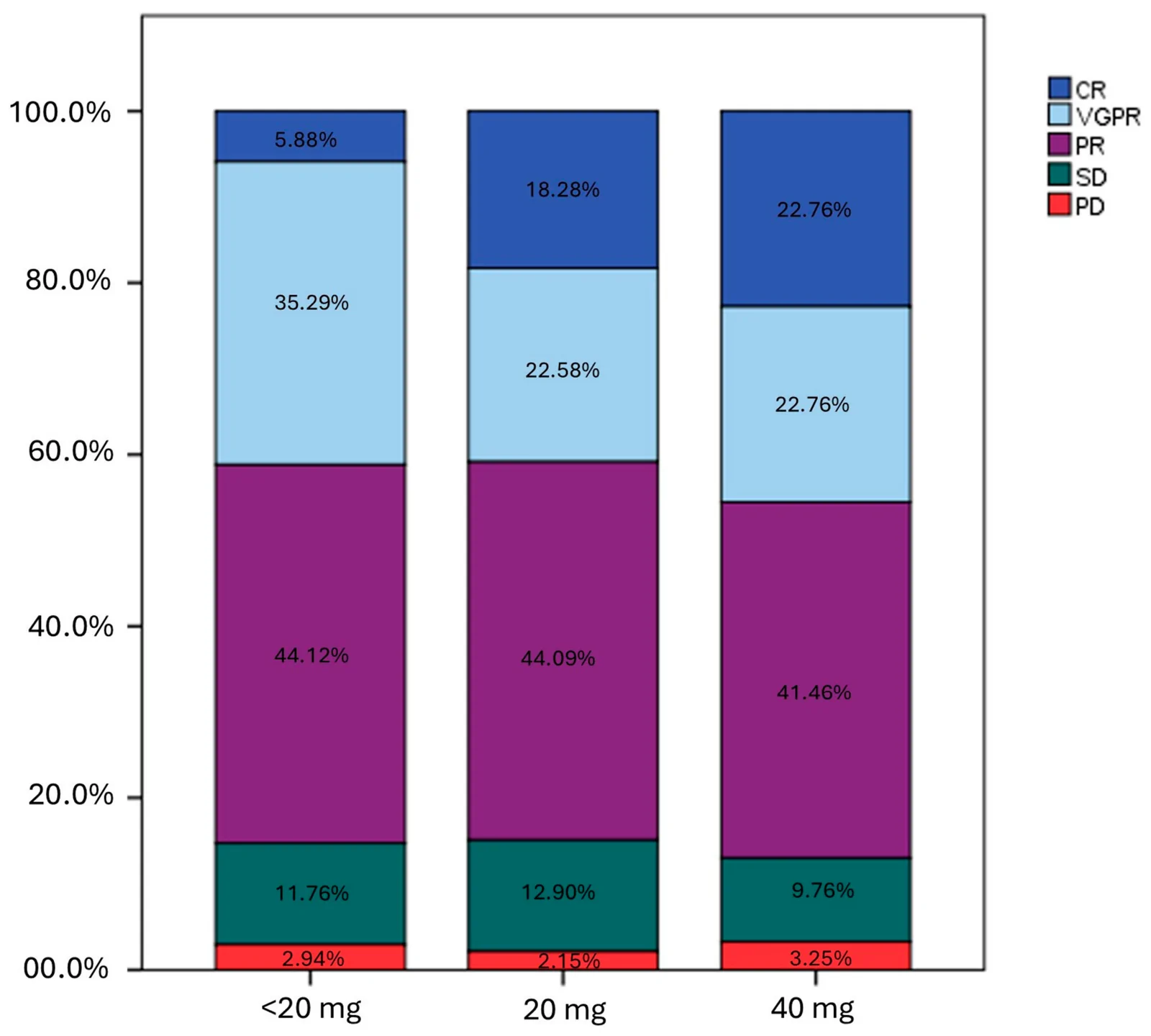
Best overall response according to dexamethasone dose.

Best overall response was evaluable in 250 of 272 patients (91.9%), including 34, 93, and 123 patients in the <20 mg, 20 mg, and 40 mg groups, respectively. We observed no statistically significant association between baseline dexamethasone dose and best overall response (χ^2^ *p* = 0.560). Specifically, complete response (CR) rates were 5.9%, 18.3%, and 22.8% in the <20 mg, 20 mg, and 40 mg cohorts, respectively. Very good partial response (VGPR) rates were 35.3%, 22.6%, and 22.8%, while partial response (PR) rates were 44.1%, 44.1%, and 41.5% across the same respective groups. These findings suggest that dexamethasone dose intensity did not significantly influence overall response rates, despite an association between reduced dosing, advancing age, and presumed increased clinical frailty. (Figure 1)

Although high-dose dexamethasone is historically considered crucial during the initial phase of therapy because of its potent anti-myeloma activity, this cohort showed no significant association between dexamethasone dose and treatment response at either the 2-month (χ^2^ *p* = 0.892) or 6-month (χ^2^ *p* = 0.097) landmarks.

### 3.3. Progression-Free Survival

**Figure 2:**
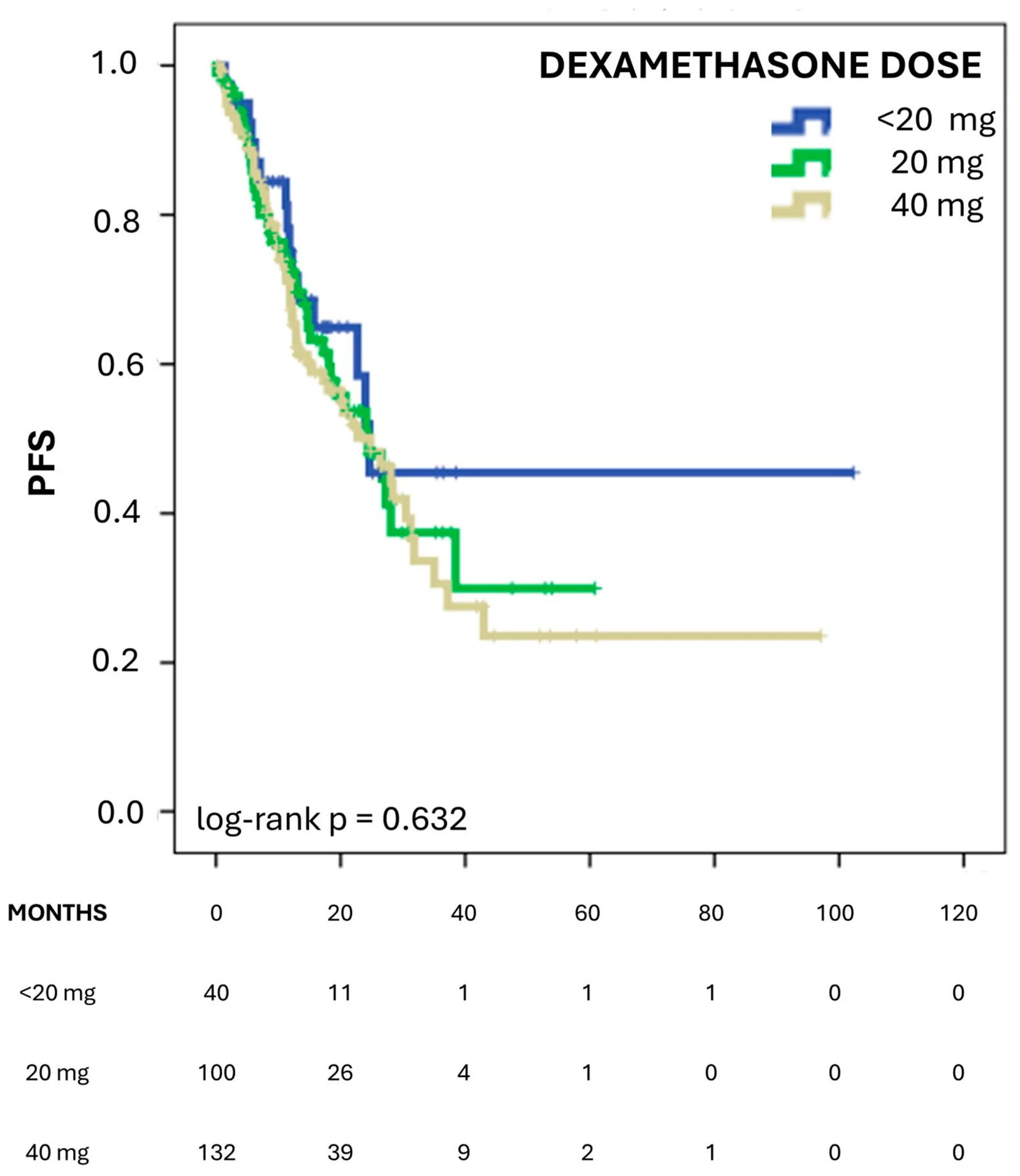
Progression-free survival according to dexamethasone dose groups with the numbers at risk displayed below the graph.

Progression-free survival was estimated using the Kaplan–Meier method (Figure 2). The median follow-up time, estimated using the reverse Kaplan–Meier method, was 19.7 months (95% CI, 17.9–21.6 months). This was shorter than the estimated median PFS. Overall, 118 PFS events were observed among 272 patients (43.4%), while 154 patients (56.6%) were censored. The numbers of PFS events were 15, 41, and 62 in the <20 mg, 20 mg, and 40 mg starting-dose groups, respectively.

Median PFS was 24.6 (95% CI NE), 24.4 (15.7–33.0), and 24.8 (17.1–32.4) months in the <20 mg, 20 mg, and 40 mg groups. The 95% confidence interval for median PFS in the <20 mg group could not be reliably estimated, in the context of only 15 observed PFS events.

No statistically significant difference was observed between the dexamethasone dose groups (log-rank *p* = 0.632). The survival curves showed substantial overlap across the three dose groups, indicating that dexamethasone dose did not significantly impact progression-free survival.

### 3.4. Cox Regression Analysis

Multivariate Cox proportional hazards regression analysis was performed to evaluate the independent effect of dexamethasone dose on progression-free survival.

The overall model was statistically significant (Omnibus test *p* = 0.019); however, dexamethasone dose was not significantly associated with progression-free survival in the multivariable Cox regression model (overall Wald test, *p* = 0.306). Compared to the reference dose category, dose-reduced groups did not demonstrate a significantly increased risk of progression or death (Category 1: HR = 0.82, 95% CI: 0.38–1.78, *p* = 0.609; Category 2: HR = 0.70, 95% CI: 0.41–1.21, *p* = 0.203). Similarly, ISS stage at diagnosis was not independently associated with PFS when compared to Stage III (Stage I: HR = 1.02, 95% CI: 0.54–1.90, *p* = 0.956; Stage II: HR = 0.64, 95% CI: 0.32–1.26, *p* = 0.198). The absence of severe renal impairment (GFR ≥ 30 mL/min) was not significantly associated with PFS compared with GFR < 30 mL/min (HR = 0.51, 95% CI: 0.24–1.11, *p* = 0.091). The detailed hazard ratios, 95% confidence intervals, and *p*-values for all variables included in the model are presented in Table 2. Overall, these findings suggest that lower dexamethasone dose did not adversely affect PFS.

### 3.5. Cytogenetic Risk Analysis

**Figure 3:**
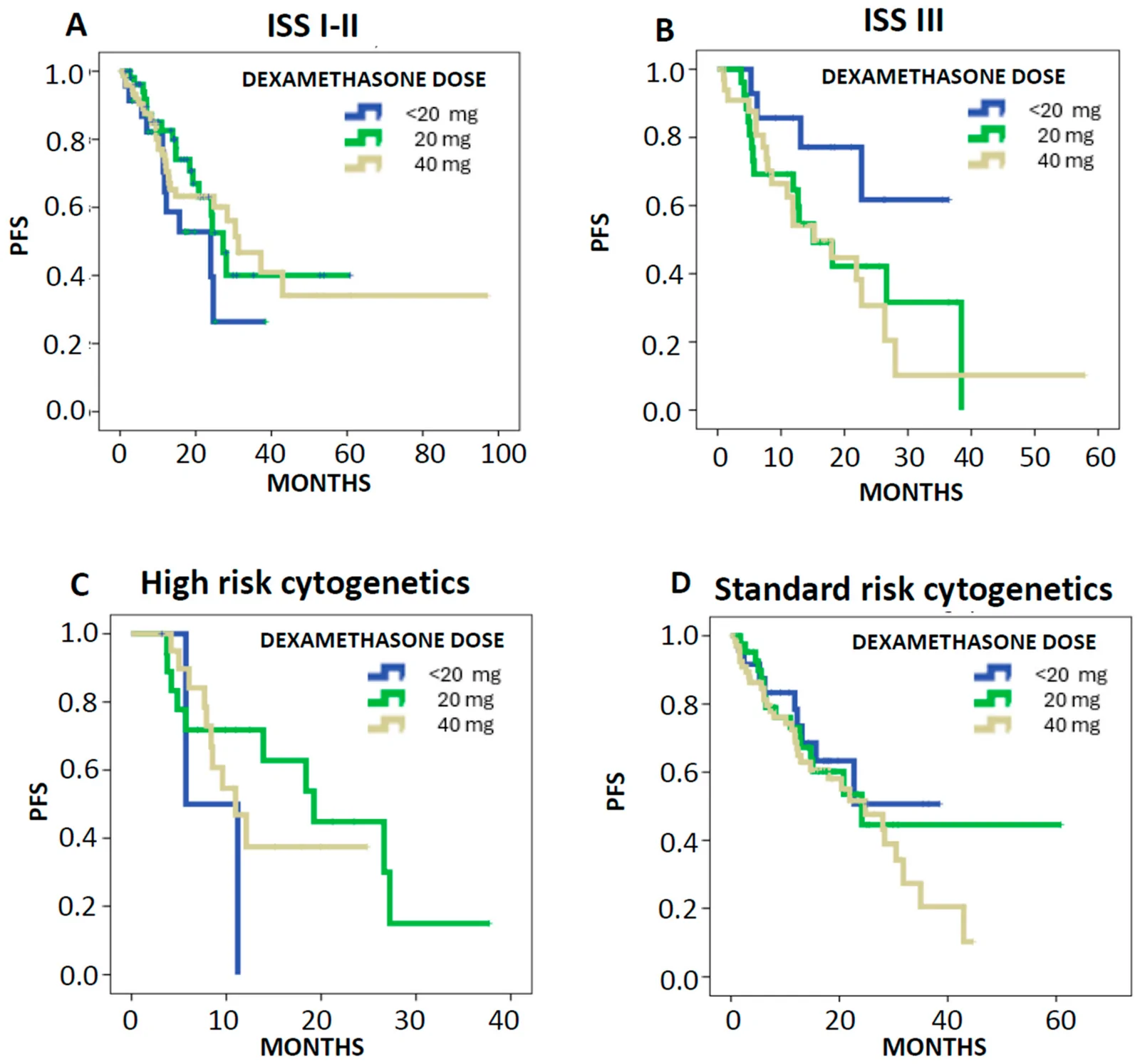
Progression-free survival according to dexamethasone dose in ISS and cytogenetic risk subgroups (**A**). ISS stage I–II, *p* = 0.345; (**B**) ISS stage III, *p* = 0.128; (**C**) high-risk cytogenetics, *p* = 0.312; (**D**) normal risk cytogenetics, *p* = 0.555. Abbreviations: PFS, Progression-Free Survival; ISS, International Staging System.

Cytogenetic data were available for 179 of the 272 patients (65.8%), high-risk abnormalities included del(17p), t(4;14), t(14;16), and 1q amplification. Different genetic variants may respond in different ways, and the cells may have different or fewer steroid receptors; hence, we tested their interaction with survival both individually and also merged as part of the high-risk category [21]. Median PFS was shorter in high-risk patients than in standard-risk patients (13.9 vs. 24.1 months), although this difference reached only borderline significance (*p* = 0.061). The negative findings in the high-risk subgroup should be interpreted cautiously because of the limited sample size. Dexamethasone dose categories did not have a significant effect on survival in any of the cytogenetic subgroups. No significant difference in PFS was observed between dexamethasone groups in the high-risk cytogenetic subgroup (log-rank *p* = 0.312) (Figure 3).

### 3.6. Adverse Events

The occurrence of important adverse events (AEs) is shown in Table 3. AEs were documented in approximately one-third of the patients across all three cohorts, with the vast majority classified as mild-to-moderate in severity. The common adverse events were infection, bone marrow suppression, and diarrhea. Despite the inherent limitations associated with the retrospective collection of toxicity data, the overall distribution of adverse event grades did not differ significantly between the dosing groups (*p* = 0.086). Although not statistically significant, higher-grade toxicities occurred numerically more frequently in the 40 mg cohort.

## 4. Discussion

Despite a compelling and ongoing clinical need, investigations addressing this therapeutic dilemma remained sparse for a long time. To date, the current body of literature relies heavily on retrospective analyses, highlighting the persistent need for robust, multicenter, real-world data to guide clinical practice. The primary objective of this large, retrospective study was to demonstrate that dexamethasone dose attenuation does not compromise clinical outcomes in patients with multiple myeloma.

In a retrospective analysis, Banerjee et al. evaluated data from 541 patients enrolled in two large Southwest Oncology Group (SWOG) clinical trials—SWOG 0777 (comparing lenalidomide–dexamethasone [Rd] versus bortezomib–lenalidomide–dexamethasone [VRd]) and SWOG 1211 (evaluating VRd versus elotuzumab-VRd)—specifically investigating the impact of corticosteroid dose reductions. In these protocols, the planned initial weekly dose was 40 mg, with de-escalation to 20 mg or 12 mg permitted only upon the occurrence of Grade 3 or higher toxicities. Notably, despite these dose reductions being triggered by severe, treatment-related AEs—which might clinically surrogate for a more vulnerable patient subset—the attenuated dexamethasone doses did not adversely affect PFS or overall survival (OS). The authors concluded that although treatment-limiting toxicities often require dexamethasone dose modifications in routine clinical practice, dose reductions do not compromise long-term survival outcomes [22].

The only prospective randomized study examining dexamethasone dose reduction in the era of novel agents is from Larocca et al. This open-label, phase III clinical trial evaluated discontinuation of dexamethasone versus continued lenalidomide–dexamethasone treatment after nine cycles of induction therapy in intermediate-fit patients with newly diagnosed multiple myeloma. Median progression-free survival was 20.2 and 18.3 months, respectively (*p* = 0.16). The study demonstrated that dropping dexamethasone after a successful nine-month induction phase was not associated with a statistically significant difference in progression-free survival while significantly reducing toxicity and improving tolerability [15].

In the present study, dexamethasone dose intensity was not associated with progression-free survival (PFS) in either univariate or multivariate analyses, suggesting that baseline corticosteroid dosing is not an independent predictor of clinical outcomes. This lack of association remained consistent across all prespecified subgroup analyses, including the subset of patients presenting with high-risk cytogenetic abnormalities. However, cytogenetic information was not available for all patients, limiting the interpretation of subgroup analyses. Several underlying mechanisms may account for these observations. First, in modern, highly active anti-myeloma regimens, high-dose dexamethasone may no longer drive cytoreduction, in contrast to historical, conventional chemotherapy-based combinations in which corticosteroids bore the principal therapeutic burden. This shift is likely to become even more pronounced as frontline quadruplet regimens become more widely adopted [23]. Second, any incremental benefit from higher corticosteroid doses may be offset by their well-documented systemic toxicities.

The clinical implications of our findings are significant. Reducing dexamethasone dose may decrease treatment-related toxicity without compromising efficacy, which is particularly relevant for older and frail patients, especially in the context of anti-CD38 antibody-based treatment. Therefore, prospective studies are warranted to confirm these findings and optimize steroid-sparing strategies.

Recently, the phase 3 IFM 2020-05/BENEFIT study, which randomized patients between isatuximab-Rd and isatuximab-VRd, utilized 20 mg dexamethasone weekly from treatment onset regardless of age, proving feasibility of the lower dose but not providing evidence of non-inferiority in comparison with the standard 40 mg weekly dose. The estimated 24-month progression-free survival rates were 80.0% and 85.2% in the IsaRd and Isa-VRd groups, respectively, whereas the IMROZ study reported a five-year progression-free survival rate of 63.2%. However, these results are not directly comparable because of differences in patient populations, treatment regimens, and follow-up duration [24].

There are two prospective studies testing the feasibility of stopping steroids after two cycles of modern anti-CD38-based triplet or quadruplet therapies. The phase III IFM2017-03 trial compared daratumumab plus lenalidomide, with dexamethasone limited to the first two cycles, against continuous lenalidomide–dexamethasone in frail patients with newly diagnosed multiple myeloma. Median PFS was 53.4 versus 22.5 months, respectively. However, because daratumumab was included only in the dexamethasone-sparing arm, the independent effect of dexamethasone discontinuation cannot be determined [25]. The similarly designed phase 2 REST study addressed the same question with the isatuximab-VRD combination in 51 elderly patients, using 20 mg dexamethasone for 2 cycles and then omitting it completely. The ORR was 100%, MRD-negative complete response was observed in 37% [26]. However, these combinations involve different treatment settings and may alter the relative contribution of dexamethasone to efficacy and toxicity but we think that if dexamethasone dose reduction was possible without jeopardizing the outcome when the effectivity of treatment was depending on only two active agents, it is even more true now, when we use quadruplets where the effectivity relies on 4 drugs.

A primary limitation of our study is the lack of objective clinical frailty data. Because frailty status is one of the main determinants of dexamethasone dose selection in routine clinical practice, residual confounding by indication cannot be excluded [27]. Standardized frailty scores were not systematically captured at the time of treatment initiation, and calculating these indices retrospectively remains highly imprecise, therefore patient age served as the sole surrogate marker for physical frailty. ECOG performance status and comorbidity data were not systematically available, limiting our ability to account for differences in patient fitness and potential confounding by indication [28].

Crucially, however, our data demonstrated no significant disparities in any clinical outcome measures across the dexamethasone dosing cohorts, despite the baseline age being significantly higher in the lower-dose groups.

Another confounding factor that is inherently difficult to quantify is the clinical intent behind dose modification. While reduced frontline dexamethasone dosing is often prompted by advanced age or baseline comorbidities, it may also reflect a more nuanced, individualized, patient-centric clinical assessment [29,30]. Consequently, proactive dose attenuation may act as a surrogate marker for superior, personalized supportive care, which could inherently optimize patient compliance and mitigate treatment-limiting toxicities.

The overall distribution of adverse event grades did not differ significantly between groups (*p* = 0.086). However, grade 3–5 AEs were numerically more frequent in patients receiving dexamethasone dose of 40 mg weekly (14.4%) compared with those receiving 20 mg (6.0%) or <20 mg (2.5%). Baseline differences between treatment groups, and potential variability in adverse event reporting across centers limit the interpretation of these findings.

The incomplete availability of cytogenetic data, which were missing for 93 patients (34.2%), reduced the sample size of the cytogenetic subgroup analyses.

## 5. Conclusions

In this lenalidomide–dexamethasone combination-therapy study, lower dexamethasone doses were not associated with worse progression-free survival, treatment response, or adverse events. No significant difference in progression-free survival was observed between dose groups in high-risk and cytogenetic patients either.

Our findings suggest that lower dexamethasone doses may be a better option, especially in older patients, without reducing treatment efficacy.

## Figures and Tables

**Table 1:** Patient characteristics.

	Weekly Dexamethasone Dose			Overall	*p*-Value
	<20 mg (*n* = 40)	20 mg (*n* = 100)	40 mg (*n* = 132)	*n* = 272	
Male/female	20/20	51/49	67/65	138/134	0.994
Median age (years)	69	68	62	65	<0.001
ISS stage at diagnosis					0.723
ISS I	8/37 (21.6%)	28/84 (33.3%)	34/110 (30.9%)	70/231 (30.3%)	
ISS II	15/37 (40.5%)	28/84 (33.3%)	42/110 (38.2%)	85/231 (36.8%)	
ISS III	14/37 (37.8%)	28/84 (33.3%)	34/110 (30.9%)	76/231 (32.9%)	
High-risk cytogenetics (n/N, %)	3/27 (11.1%)	18/62 (29.0%)	21/90 (23.3%)	42/179 (23.5%)	0.186
Renal impairment (GFR < 30 mL/min) (n/N, %)	7/40 (17.5%)	12/100 (12.0%)	15/131 (11.5%)	34/271 (12.5%)	0.587
number of prior lines, median (range)	2 (1–4)	2 (1–4)	2 (1–4)	2 (1–4)	0.953
prior ASCT (n/N, %)	11/40 (27.5%)	35/100 (35.0%)	77/131 (58.8%)	123/271 (45.4%)	<0.001

Abbreviations: ISS, International Staging System; ASCT, Autologous Stem Cell Transplant; GFR, Glomerular Filtration Rate.

**Table 2:** Multivariable Cox proportional hazards regression analysis.

Variable	Category	Hazard Ratio	95% CI	*p*-Value
Dexamethasone dose	Overall effect	-	-	0.306
	<20 mg	0.82	0.38–1.78	0.609
	20 mg	0.70	0.41–1.21	0.203
	40 mg *(Reference)*	1.00	-	-
Renal impairment	GFR ≥ 30 mL/min *(Reference)*	1.00	-	-
	GFR < 30 mL/min	0.51	0.24–1.11	0.091
ISS stage at diagnosis	Stage I	1.02	0.54–1.90	0.956
	Stage II	0.64	0.32–1.26	0.198
	Stage III *(Reference)*	1.00	-	-

**Table 3:** Safety analysis. The distribution of AEs by severity grade across dose groups (χ^2^ *p* = 0.086).

AE Grade	<20 mg	20 mg	40 mg
no toxicity recorded	28/40 (70%)	75/100 (75%)	97/132 (73.5%)
Grade 1	5/40 (12.5%)	4/100 (4.0%)	9/132 (6.8%)
Grade 2	6/40 (15.0%)	15/100 (15.0%)	7/132 (5.3%)
Grade 3	1/40 (2.5%)	3/100 (3%)	12/132 (9.1%)
Grade 4	0/40 (0%)	2/100 (2%)	5/132 (3.8%)
Grade 5	0/40 (0%)	1/100 (1%)	2/132 (1.5%)

## Data Availability

The de-identified participant data, study protocol, and statistical analysis plan underlying this study’s findings are not publicly available due to patient privacy and ethical restrictions. However, the corresponding author will make the data available upon reasonable request from qualified researchers.

## Abbreviations

The following abbreviations are used in this manuscript:

AE	Adverse Event
ASCT	Autologous Stem Cell Transplant
CI	Confidence Interval
CR	Complete Response
FISH	Fluorescence In Situ Hybridization
GFR	Glomerular Filtration Rate
HR	Hazard Ratio
IMiDs	Immunomodulatory Drugs
IMWG	International Myeloma Working Group
ISS	International Staging System
NE	Not Estimable
MRD	Minimal Residual Disease
ORR	Overall Response Rate
OS	Overall Survival
PFS	Progression-Free Survival
PIs	Proteasome Inhibitors
PR	Partial Remission
Rd	Lenalidomide-dexamethasone
SD	Stable Disease
VGPR	Very Good Partial Remission
VRd	Bortezomib-Lenalidomide-Dexamethasone

## References

[1] Malard F., Neri P., Bahlis N.J., Terpos E., Moukalled N., Hungria V.T.M., Manier S., Mohty M. (2024). Multiple myeloma. Nat. Rev. Dis. Primers.

[2] National Health Insurance Fund of Hungary (NEAK) A Myeloma Multiplex Kezelésének Finanszírozási Eljárásrendje. https://www.neak.gov.hu/pfile/file?path=/letoltheto/ATFO_dok/2026/fin_elj_myeloma-multiplex-_neak_publikalhato&inline=true.

[3] Attal M., Lauwers-Cances V., Hulin C., Leleu X., Caillot D., Escoffre M., Arnulf B., Macro M., Belhadj K., Garderet L. (2017). Lenalidomide, bortezomib, and dexamethasone with transplantation for myeloma. N. Engl. J. Med..

[4] Kumar S.K., Dispenzieri A., Lacy M.Q., Gertz M.A., Buadi F.K., Pandey S., Kapoor P., Dingli D., Hayman S.R., Leung N. (2014). Continued improvement in survival in multiple myeloma: Changes in early mortality and outcomes in older patients. Leukemia.

[5] Moreau P., Kumar S.K., San Miguel J., Davies F., Zamagni E., Bahlis N., Ludwig H., Mikhael J., Terpos E., Schjesvold F. (2021). Treatment of relapsed and refractory multiple myeloma: Recommendations from the International Myeloma Working Group. Lancet Oncol..

[6] Banerjee R., King T., Faiman B., Harding S., Rosenberg A.S., Sanchorawala V., Mikhael J.R., Cowan A.J. (2026). Past, present, and future of dexamethasone in multiple myeloma and AL amyloidosis. J. Clin. Oncol..

[7] Alexanian R., Barlogie B., Dixon D. (1986). High-dose glucocorticoid treatment of resistant myeloma. Ann. Intern. Med..

[8] Rajkumar S.V., Jacobus S., Callander N.S., Fonseca R., Vesole D.H., Williams M.E., Abonour R., Siegel D.S., Katz M., Greipp P.R. (2010). Lenalidomide plus high-dose dexamethasone versus lenalidomide plus low-dose dexamethasone as initial therapy for newly diagnosed multiple myeloma: An open-label randomised controlled trial. Lancet Oncol..

[9] Moreau P., San Miguel J., Sonneveld P., Mateos M.V., Zamagni E., Avet-Loiseau H., Hajek R., Dimopoulos M.A., Ludwig H., Einsele H. (2017). Multiple myeloma: ESMO Clinical Practice Guidelines for diagnosis, treatment and follow-up. Ann. Oncol..

[10] Kumar S.K., Callander N.S., Adekola K., Anderson L., Baljevic M., Campagnaro E., Castillo J.J., Chandler J.C., Costello C., Efebera Y. (2020). Multiple myeloma, version 3.2021, NCCN Clinical Practice Guidelines in Oncology. J. Natl. Compr. Cancer Netw..

[11] Dimopoulos M.A., Moreau P., Terpos E., Mateos M.V., Zweegman S., Cook G., Delforge M., Hajek R., Schjesvold F.H., Cavo M. (2021). Multiple myeloma: EHA-ESMO Clinical Practice Guidelines for diagnosis, treatment and follow-up. Ann. Oncol..

[12] Mikhael J., Ismaila N., Cheung M.C., Costello C., Dhodapkar M.V., Kumar S., Lacy M., Lipe B., Little R.F., Nikonova A. (2019). Treatment of Multiple Myeloma: ASCO and CCO Joint Clinical Practice Guideline. J. Clin. Oncol..

[13] Hulin C., Belch A., Shustik C., Petrucci M.T., Dührsen U., Lu J., Song K., Rodon P., Pégourié B., Garderet L. (2016). Updated outcomes and impact of age with lenalidomide and low-dose dexamethasone or melphalan, prednisone, and thalidomide in the randomized, phase III FIRST trial. J. Clin. Oncol..

[14] Mateos M.V., Leleu X., Palumbo A., San Miguel J.F. (2014). Initial treatment of transplant-ineligible patients in multiple myeloma. Expert Rev. Hematol..

[15] Larocca A., Bonello F., Gaidano G., D’Agostino M., Offidani M., Cascavilla N., Capra A., Benevolo G., Tosi P., Galli M. (2021). Dose/schedule-adjusted Rd-R vs continuous Rd for elderly, intermediate-fit patients with newly diagnosed multiple myeloma. Blood.

[16] McKenzie F., Gazzé G., Hewitt J., Kolm K., Pollock D., Rowland S., Crosbie T. (2022). Canadian perspectives in multiple myeloma on the use of steroids in clinical practice based on patient and healthcare provider interviews. Front. Oncol..

[17] Sonneveld P., Broijl A. (2016). Treatment of relapsed and refractory multiple myeloma. Haematologica.

[18] Kyle R.A., Rajkumar S.V. (2009). Criteria for diagnosis, staging, risk stratification and response assessment of multiple myeloma. Leukemia.

[19] Schemper M., Smith T.L. (1996). A note on quantifying follow-up in studies of failure time. Control. Clin. Trials.

[20] Durie B.G.M., Harousseau J.L., Miguel J.S., Bladé J., Barlogie B., Anderson K., Gertz M., Dimopoulos M., Westin J., Sonneveld P. (2006). International uniform response criteria for multiple myeloma. Leukemia.

[21] Kervoëlen C., Ménoret E., Gomez-Bougie P., Bataille R., Godon C., Marionneau-Lambot S., Moreau P., Pellat-Deceunynck C., Amiot M. (2015). Dexamethasone-induced cell death is restricted to specific molecular subgroups of multiple myeloma. Oncotarget.

[22] Banerjee R., Sexton R., Cowan A.J., Rosenberg A.S., Ailawadhi S., Rajkumar S.V., Kumar S., Dispenzieri A., Lonial S., Durie B.G.M. (2025). Dexamethasone dose intensity does not impact outcomes in newly diagnosed multiple myeloma: A secondary SWOG analysis. Blood.

[23] Gay F., Engelhardt M., Terpos E., Wäsch R., Giaccone L., Auner H.W., Caers J., Gramatzki M., van de Donk N., Oliva S. (2018). From transplant to novel cellular therapies in multiple myeloma: European Myeloma Network guidelines and future perspectives. Haematologica.

[24] Leleu X., Hulin C., Lambert J., Bobin A., Perrot A., Karlin L., Roussel M., Montes L., Cherel B., Chalopin T. (2024). Isatuximab, lenalidomide, dexamethasone and bortezomib in transplant-ineligible multiple myeloma: The randomized phase 3 BENEFIT trial. Nat. Med..

[25] Manier S., Lambert J., Hulin C., Macro M., Laribi K., Araujo C., Pica G.M., Touzeau C., Godmer P., Slama B. (2025). Safety and efficacy of a dexamethasone-sparing regimen with daratumumab and lenalidomide in patients with frailty and newly diagnosed multiple myeloma (IFM2017-03): A phase 3, open-label, multicentre, randomised, controlled trial. Lancet Oncol..

[26] Askeland F.B., Haukås E., Slørdahl T.S., Klostergaard A., Alexandersen T., Osnes L., Lysén A., Abdollahi P., Nielsen L.K., Hermansen E. (2025). Isatuximab, bortezomib, lenalidomide, and limited dexamethasone in patients with transplant-ineligible multiple myeloma (REST): A multicentre, single-arm, phase 2 trial. Lancet Haematol..

[27] Palumbo A., Bringhen S., Mateos M.V., Larocca A., Facon T., Kumar S.K., Offidani M., McCarthy P., Evangelista A., Lonial S. (2015). Geriatric assessment predicts survival and toxicities in elderly myeloma patients: An International Myeloma Working Group report. Blood.

[28] Lee H.S., Kim K., Lee J.J., Yoon S.S., Bang S.M., Kim J.S., Eom H.S., Yoon D.H., Lee Y., Shin H.J. (2021). Clinical impact of frailty on treatment outcomes of elderly patients with relapsed and/or refractory multiple myeloma treated with lenalidomide plus dexamethasone. Int. J. Hematol..

[29] Wildes T.M., Anderson K.C. (2018). Approach to the treatment of the older, unfit patient with myeloma from diagnosis to relapse: Perspectives of a US hematologist and a geriatric hematologist. Hematol. Am. Soc. Hematol. Educ. Program.

[30] Larocca A., Palumbo A. (2015). How I treat fragile myeloma patients. Blood.

